# Comparative Phytochemical Characterization, Biological Activities and Safety Assessment of *Salvia pratensis* L. and *Salvia sclarea* L.

**DOI:** 10.3390/plants15071038

**Published:** 2026-03-27

**Authors:** Mariana Panţuroiu, Mona Luciana Gălăţanu, Sorina Nicoleta Voicu, Emilia Pănuş, Luiza Mădălina Cima, Andrei Biţă, Carmen Marinela Mihăilescu, Carmen-Elisabeta Manea, Adina Turcu-Știolică, Manuel Ovidiu Amzoiu, Mirela Claudia Rîmbu, Daniel Cord, Ion Mircioiu

**Affiliations:** 1 Faculty of Pharmacy, Titu Maiorescu University, Sincai Boulevard, No. 16, 040314 Bucharest, Romania; mariana.panturoiu@prof.utm.ro (M.P.); luiza.cima@prof.utm.ro (L.M.C.); carmen.mihailescu@prof.utm.ro (C.M.M.); carmen.manea@prof.utm.ro (C.-E.M.); mirela.rimbu@prof.utm.ro (M.C.R.); daniel.cord@prof.utm.ro (D.C.); ion.mircioiu@prof.utm.ro (I.M.); 2 Department of Biochemistry and Molecular Biology, Faculty of Biology, University of Bucharest, Splaiul Independenței 91-95, 050095 Bucharest, Romania; 3 Department of Biochemistry, Faculty of Medicine, Ovidius University of Constanta, Universitatii Street, No. 1, 900470 Constanta, Romania; temilia2@yahoo.com; 4 Microbiology and Molecular Biology Laboratory, Public Health Directorate Constanta, 89 Nicolae Iorga Street, 900587 Constanta, Romania; 5 Faculty of Pharmacy, University of Medicine and Pharmacy of Craiova, 200349 Craiova, Romania; andrei.bita@umfcv.ro (A.B.); adina.turcu@umfcv.ro (A.T.-Ș.); manuel.amzoiu@umfcv.ro (M.O.A.); 6 National Institute for Research and Development in Microtechnologies (IMT Bucharest), 072996 Bucharest, Romania; 7 Horia Hulubei National Institute for R&D in Physics and Nuclear Engineering (IFIN-HH), 077125 Magurele, Romania

**Keywords:** *Salvia sclarea*, *Salvia pratensis*, phenolic acids, essential oil composition, pesticide residues, heavy metals, antioxidant activity, antimicrobial activity, biocompatibility

## Abstract

This study provides a comparative evaluation of two *Salvia* species, the widely cultivated *Salvia sclarea* L. and the comparatively underexplored wild species *Salvia pratensis* L., integrating phytochemical profiling, chemical safety assessment, and biological activity investigation. Dried hydroethanolic extracts and essential oils obtained from aerial parts were analysed. HPLC–PDA analysis revealed distinct phenolic acid profiles, with *S. sclarea* characterized by higher levels of rosmarinic and protocatechuic acids, whereas *S. pratensis* contained greater amounts of hydroxycinnamic acids such as caffeic, p-coumaric, and ferulic acids. The total phenolic content was higher in *S. pratensis* (79.22 mg GAE/g dry extract) than in *S. sclarea* (52.50 mg GAE/g). GC–MS analysis showed that the essential oil of *S. sclarea* was dominated by oxygenated monoterpenes, mainly linalyl acetate and linalool, while *S. pratensis* exhibited a linalool-rich profile accompanied by sesquiterpene derivatives. Chemical safety assessment indicated minimal contamination, with pesticide residues detected only in *S. sclarea* at levels below regulatory limits and low concentrations of cadmium and lead in both species. The extracts showed strong antioxidant activity (DPPH IC_50_ values of 6.67 µg/mL for *S. sclarea* and 3.16 µg/mL for *S. pratensis*) and moderate broad-spectrum antimicrobial activity (MIC 312.5–2500 µg/mL). In vitro assays on HEK 293 and HaCaT cells confirmed low cytotoxicity, with no evidence of membrane damage or pro-inflammatory effects. Overall, the results highlight the significant bioactive potential of the less studied *S. pratensis*, demonstrating that this wild species represents a promising alternative source of natural antioxidant and antimicrobial compounds comparable to the widely cultivated *S. sclarea*.

## 1. Introduction

The genus *Salvia* belongs to the Lamiaceae family, order Lamiales, and comprises approximately 1000 species of aromatic shrubs and herbaceous plants distributed worldwide, predominantly in tropical and temperate regions. Species of this genus are well known for their phytochemical diversity and pharmacological significance, being rich in secondary metabolites such as phenolic acids, flavonoids, diterpenoids, and volatile terpenoids [1,2]. These compounds contribute to the broad spectrum of biological activities reported for *Salvia* species, including antioxidant, anti-inflammatory, antimicrobial, antidiabetic, neuroprotective, and cytotoxic effects [1,3]. Consequently, numerous *Salvia* species are widely used in traditional medicine, as well as in the food, pharmaceutical, and cosmetic industries.

Among the *Salvia* species growing in Romania, *Salvia sclarea* L. (clary sage) is a widely cultivated plant valued for its aromatic, medicinal, and industrial applications. Traditionally, the species has been used for the treatment of various ailments, including muscular pain, digestive disorders, skin irritations, excessive sweating, gingivitis, colds, and throat infections [2]. In recent years, *S. sclarea* has attracted considerable scientific attention, and numerous studies have confirmed a broad spectrum of biological activities, including antioxidant [1,4,5], antibacterial [1,6], antifungal [1,4,6], antidiabetic [5,7], anxiolytic [8], neuroprotective [5,9,10], and cytotoxic or antiproliferative effects [1,4,8,11,12]. Owing to its essential oil composition, *S. sclarea* is also widely utilised in cosmetic products and perfumery [13,14]. Phytochemical investigations have revealed a complex chemical profile in the aerial parts of *S. sclarea*, including flavonoids (such as luteolin, apigenin, cirsimaritin, genkwanin, and their derivatives), phenolic acids (caffeic acid, rosmarinic acid, caffeoylthreonic acid, salvianic acid, salvianolic acid B), diterpenes (hydroxycarnosic acid I, sclareol, manool, salvipisone, ferruginol, microstegiol, candidissiol, 7-oxoroyleanone, A and B), as well as fatty acids (hydroxyoxooctadecadienoic acid, hydroxyoctadecatrienoic acid, hydroxyoctadecadienoic acid), as well as sugars, and other specialised metabolites [1,15,16]. The seeds contain a lectin (SSL) capable of agglutinating Tn erythrocytes and recognising Tn antigens expressed in certain human cancer cell types [17,18], as well as fatty acids and phenolic compounds [19]. The essential oil of *S. sclarea*, rich in compounds such as linalyl acetate, linalool, and geranyl acetate, is particularly valued in the cosmetic and perfumery industries [4,20,21,22,23].

In contrast, *S. pratensis* L. (meadow sage) is a comparatively understudied sage species that grows spontaneously in meadows, pastures, and along roadsides in the hilly and mountainous regions of Romania. Despite belonging to the same genus, *S. pratensis* has received considerably less scientific attention compared with cultivated *Salvia* species. Existing phytochemical studies have reported the presence of phenolic acids (rosmarinic acid, methylrosmarinate, caffeic acid, salvianolic acid A, B and H, carnosic acid, hydroxybenzoic acid, caffeoylthreonic acid), flavonoids (luteolin, apigenin, cirsimaritin, genkwanin), diterpenoids (rosmadial, rosmanol III, abietane diterpenes), triterpenoids, fatty acids, sugars, and organic acids in the aerial parts and roots of the plant [24,25,26]. Moreover, recent investigations have indicated that extracts obtained from *S. pratensis* may exhibit antioxidant [24,27], antibacterial [24], antidiabetic, neuroprotective [5], and cytotoxic activities against certain cancer cell lines [24,28]. The essential oil of *S. pratensis* has been reported to contain predominantly sesquiterpene hydrocarbons, accompanied by smaller amounts of monoterpenes and other volatile compounds [23,29,30]. However, compared with extensively investigated cultivated species such as *S. sclarea*, the phytochemical profile and biological potential of *S. pratensis* remain relatively underexplored, particularly in integrated studies combining phytochemical characterisation, biological activity evaluation, and safety assessment. Considering the contrasting ecological and agronomic status of the two species, a comparative investigation may provide valuable insights into how domestication, cultivation practices, and environmental factors influence the phytochemical composition and bioactive potential of *Salvia* species. While cultivated plants such as *S. sclarea* have been extensively studied and commercially exploited, wild species such as *S. pratensis* may represent an underutilised source of biologically active compounds.

In addition to their phytochemical richness and biological relevance, *Salvia* species intended for medicinal, food, or cosmetic applications require careful evaluation of chemical safety. Medicinal and aromatic plants may be exposed to contaminants such as pesticide residues and heavy metals through agricultural practices or environmental sources, including soil, irrigation water, and atmospheric deposition. Although these compounds are not intrinsic constituents of plant secondary metabolism, their presence may influence plant physiology and therefore requires careful evaluation to ensure quality control, regulatory compliance, and the safe use of plant-derived materials.

In this context, the present study aims to provide a comprehensive comparative characterisation of *S. sclarea* and *S. pratensis* by integrating phytochemical profiling with the evaluation of their biological activities. The total phenolic content of dried hydroethanolic extracts was determined using spectrophotometric methods, while major phenolic acids were identified and quantified by HPLC–PDA analysis. In parallel, the chemical composition of the essential oils was characterised by GC–MS. The biological potential of the extracts was further assessed through in vitro antioxidant (DPPH, FRAP, ABTS), antimicrobial, and biocompatibility assays on human renal cells and keratinocytes. Furthermore, a chemical safety evaluation was performed by determining pesticide residues and selected heavy metals, enabling an integrated assessment of the phytochemical profile, biological activity, and safety of the investigated *Salvia* species.

## 2. Results

### 2.1. HPLC–PDA Identification and Quantification of Phenolic Acids

**Figure 1:**
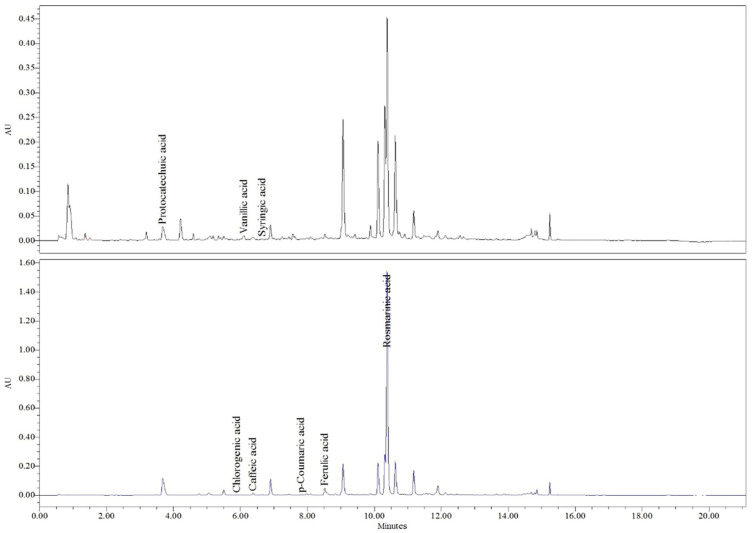
HPLC chromatogram of *S. sclarea* at 265 nm and 325 nm.

**Figure 2:**
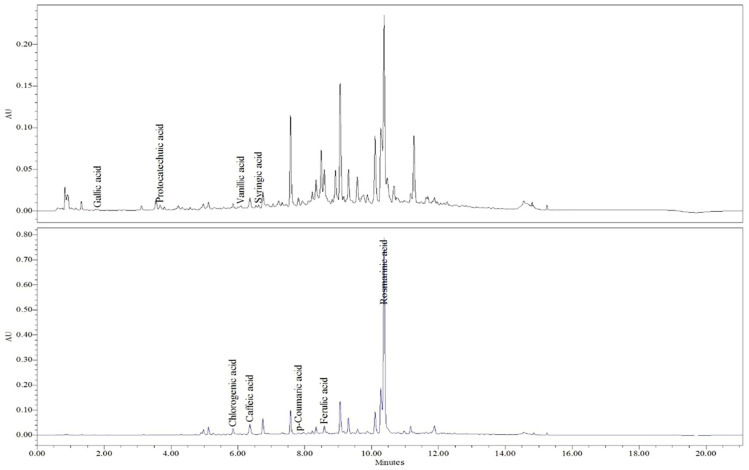
HPLC chromatogram of *S. pratensis* at 265 nm and 325 nm.

HPLC–PDA analysis enabled the identification and quantification of eight phenolic acids in the extracts of *S. sclarea* and *S. pratensis* (Table 1). Compounds were identified by comparison with authentic standards based on their retention times and UV spectra, as illustrated in the representative chromatograms presented in Figure 1 and Figure 2, respectively.

Both *Salvia* species exhibited a similar qualitative profile of phenolic acids; however, notable quantitative differences were observed. Rosmarinic acid was the predominant phenolic compound in both extracts, with higher concentrations in *S. sclarea* (181.30 ± 5.26 μg/g d.w.) than in *S. pratensis* (92.02 ± 3.88 μg/g d.w.). Protocatechuic acid was also present at a markedly higher level in *S. sclarea* (44.17 ± 1.59 μg/g d.w.), whereas only trace amounts were detected in *S. pratensis* (2.72 ± 0.01 μg/g d.w.).

In contrast, *S. pratensis* contained higher amounts of several hydroxycinnamic acids, including caffeic acid (31.54 ± 0.36 μg/g d.w.), p-coumaric acid (16.32 ± 0.51 μg/g d.w.), and ferulic acid (8.20 ± 0.02 μg/g d.w.), compared to *S. sclarea*. Vanillic acid was detected in both species at relatively low concentrations, with slightly higher levels observed in *S. pratensis*. Chlorogenic and syringic acids were detected in *S. sclarea* but remained below the limit of quantification, whereas both compounds were quantified in *S. pratensis*. Overall, the quantitative distribution of phenolic acids differed between the two species, with *S. sclarea* characterised by higher levels of rosmarinic and protocatechuic acids and *S. pratensis* showing elevated concentrations of several hydroxycinnamic acids.

While HPLC–PDA analysis provided detailed information on individual phenolic acids, total phenolic content was determined in dried extracts to obtain an overall estimation of phenolic compounds relevant to the interpretation of antioxidant and antimicrobial activities.

### 2.2. Spectrophotometric Determination of Total Polyphenols

The dried hydroethanolic extract of *S. sclarea* exhibited a total phenolic content of 52.50 ± 0.03 mg GAE/g dry extract, whereas a higher value was determined for *S. pratensis*, reaching 79.22 ± 0.06 mg GAE/g dry extract. These results indicate a higher phenolic richness of the S. pratensis extract and reflect species-specific differences in phenolic composition, further investigated by HPLC analysis.

### 2.3. Hydrodistillation and GC-MS Analysis of the Essential Oil

Hydrodistillation of the aerial parts of *S. sclarea* and *S. pratensis* resulted in essential oils with markedly different yields. The essential oil yield obtained from *S. sclarea* was 0.60% (*v*/*w*), whereas *S. pratensis* yielded only 0.10% (*v*/*w*), confirming its classification as a species poor in essential oil.

**Figure 3:**
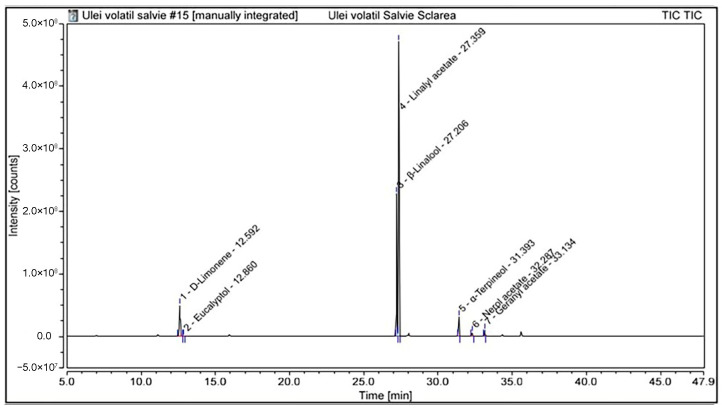
GC-MS chromatogram of *S. sclarea* essential oil.

**Figure 4:**
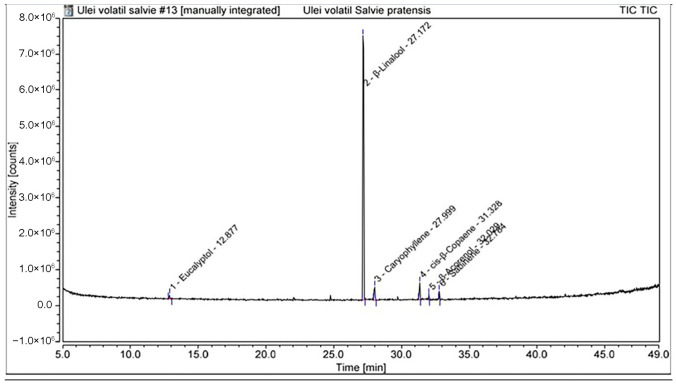
GC-MS chromatogram of *S. pratensis* essential oil.

The chemical composition of the essential oils was subsequently characterised by GC–MS analysis. The identified volatile constituents and their relative contents are summarised in Table 2, while the distribution of compounds according to their chemical classes is presented in Table 3. Representative total ion chromatograms of the essential oils from *S. sclarea* and *S. pratensis* are presented in Figure 3 and Figure 4, respectively.

The GC–MS analysis revealed distinct qualitative and quantitative differences between the essential oils of *S. sclarea* and *S. pratensis*.

The essential oil of *S. sclarea* was characterised by a clear predominance of oxygenated monoterpenes, accounting for approximately 90.7% of the total oil composition. The major constituents were linalyl acetate (58.39%) and β-linalool (26.03%). Other components were present at lower levels, including d-limonene (9.32%), α-terpineol (4.37%), geranyl acetate (1.18%), and nerol acetate (0.68%), while eucalyptol was detected only at trace levels (0.03%). Overall, the oil was dominated by esterified and free monoterpene alcohols, whereas monoterpene hydrocarbons represented only a minor fraction of the composition. In contrast, the essential oil of *S. pratensis* was strongly dominated by β-linalool (80.52%), accompanied by lower amounts of sesquiterpene hydrocarbons, mainly caryophyllene (5.81%) and cis-β-copaene (7.30%). Minor constituents included sabinene (2.84%), eucalyptol (2.27%), and β-acorenol (1.26%). Overall, compared with *S. sclarea, S. pratensis* exhibited a linalool-rich profile with a moderate contribution of sesquiterpene hydrocarbons, while oxygenated sesquiterpenes were present only in minor amounts.

Taken together, the GC–MS results highlight pronounced differences in the essential oil composition of the two *Salvia* species, with *S. sclarea* characterised by a linalyl acetate-rich profile, whereas *S. pratensis* exhibited a linalool-dominated composition accompanied by a higher relative contribution of sesquiterpene hydrocarbons.

### 2.4. Chemical Safety Assessment

To support the quality and safety evaluation of the investigated *Salvia* species, a complementary chemical safety assessment was performed, focusing on pesticide residues and selected toxic heavy metals.

#### 2.4.1. Pesticide Residues and Heavy Metal Content

Pesticide residue analysis revealed distinct contamination patterns between the two *Salvia* species. In *S.sclarea*, GC–MS/MS analysis enabled the quantification of two pyrethroid insecticides, permethrin and cypermethrin, while no additional residues above the limit of quantification (LOQ) were detected by LC–MS/MS. In contrast, no pesticide residues above the LOQ were detected in *S. pratensis* using either analytical technique. All quantified residues detected in *S. sclarea* were below the maximum residue limits established by current EU legislation (Table 4 and Table 5).

#### 2.4.2. Determination of Heavy Metals

Cadmium and lead were detected at low concentrations in both *Salvia* samples (Table 6). The highest Cd level was observed in *S. sclarea* (0.166 mg/kg), while the highest Pb concentration was found in *S. pratensis* (0.065 mg/kg). All measured values were below the maximum levels established by European regulations.

### 2.5. Biological Activity Determinations

#### 2.5.1. Antioxidant Activity

The antioxidant potential of the dried hydroethanolic extracts of *S. sclarea* and *S. pratensis* was evaluated using complementary in vitro assays, including the DPPH and ABTS radical scavenging methods and the ferric reducing antioxidant power (FRAP) assay. For the radical scavenging assays (DPPH and ABTS), all IC_50_ values were calculated based on the final concentrations of the extracts in the reaction mixture. In the DPPH assay, the IC_50_ value of *S. sclarea* was 6.67 ± 0.22 µg/mL, whereas *S. pratensis* exhibited a lower IC_50_ value of 3.16 ± 0.13 µg/mL, indicating a higher radical-scavenging activity. Vitamin C, used as a reference antioxidant, showed a markedly stronger effect, with an IC_50_ value of 0.75 ± 0.03 µg/mL under identical experimental conditions.

The ferric reducing antioxidant power (FRAP) assay further demonstrated the reducing capacity of both extracts. The dried extract of *S. pratensis* exhibited a higher antioxidant activity, with a FRAP value of 531.4 ± 12.7 µmol Fe^2+^ equivalents/g dry extract, compared with 248.2 ± 6.1 µmol Fe^2+^ equivalents/g dry extract for *S. sclarea*.

The ABTS radical scavenging assay confirmed the same trend. The extract of *S. pratensis* displayed stronger activity than *S. sclarea*, with IC_50_ values of 7.78 ± 0.27 µg/mL and 12.17 ± 0.38 µg/mL, respectively, while vitamin C exhibited an IC_50_ value of 1.94 µg/mL; all values are expressed as final concentrations in the reaction mixture.

#### 2.5.2. Antimicrobial Activity

**Figure 5:**
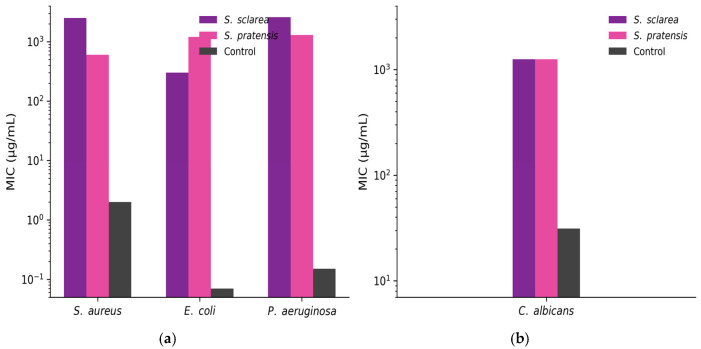
Minimum inhibitory concentrations (MIC) of dried extracts from *Salvia sclarea* and *Salvia pratensis*. (**a**) Antibacterial activity against *Staphylococcus aureus*, *Escherichia coli*, and *Pseudomonas aeruginosa*, compared with norfloxacin (positive control). (**b**) Antifungal activity against *Candida albicans,* compared with nystatin (positive control). MIC values were determined using the broth microdilution method. All experiments were performed in triplicate and yielded consistent results.

The antimicrobial activity of the *Salvia* extracts was evaluated against reference Gram-positive, Gram-negative, and yeast strains using the broth microdilution method. Both *S. pratensis* and *S. sclarea* dried extracts exhibited inhibitory activity against all tested microorganisms within the investigated concentration range, as shown in Figure 5.

The minimum inhibitory concentration (MIC) values ranged between 312.5 and 2500 µg/mL, indicating a concentration-dependent antimicrobial effect. Such MIC values are consistent with complex plant extracts, where bioactivity results from synergistic interactions among multiple phytochemicals rather than from single highly potent molecules

Overall, the extract of *S. pratensis* generally showed higher antimicrobial activity than *S. sclarea*, displaying lower MIC values against most of the tested bacterial strains.

Antibacterial effects were observed against *Staphylococcus aureus*, *Escherichia coli*, and *Pseudomonas aeruginosa*, with the lowest MIC value (312.5 µg/mL) recorded for *S. pratensis*. In contrast, *S. sclarea* typically required higher concentrations to inhibit bacterial growth.

Both extracts also demonstrated antifungal activity against *Candida albicans*, with MIC values of 1250 µg/mL. Norfloxacin, used as a positive control, exhibited markedly lower MIC values (1.95 µg/mL for *S. aureus*, 0.07 µg/mL for *E. coli*, and 0.15 µg/mL for *P. aeruginosa*), confirming the validity of the assay. Nystatin showed strong antifungal activity against *C. albicans*, with an MIC of 31.25 µg/mL. The solvent control (DMSO) did not exhibit inhibitory activity at the tested concentrations.

### 2.6. In Vitro Biocompatibility and Cytotoxicity Assessment

**Figure 6:**
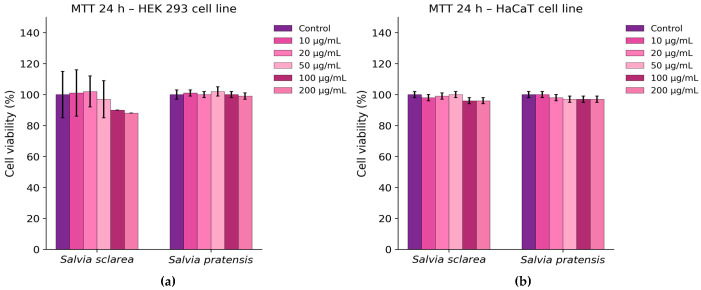
Cell viability after 24 h treatment with *Salvia* extracts (10–200 µg/mL): (**a**) HEK 293 cells; (**b**) HaCaT cells. Viability is expressed as % of control (mean ± SD). Statistical analysis was performed using two-way ANOVA followed by Dunnett’s test (*p* < 0.05 vs. control).

Cell viability assays demonstrated a generally good biocompatibility profile of both *Salvia* extracts. As shown in Figure 6a, treatment of HEK 293 cells with the extract resulted in a slight reduction in viability (approximately 15%) at the highest tested concentrations (100–200 µg/mL), whereas *S. pratensis* maintained viability levels comparable to the untreated control. In HaCaT keratinocytes, no significant changes in cell viability were observed for either extract across the tested concentration range (Figure 6b).

**Figure 7:**
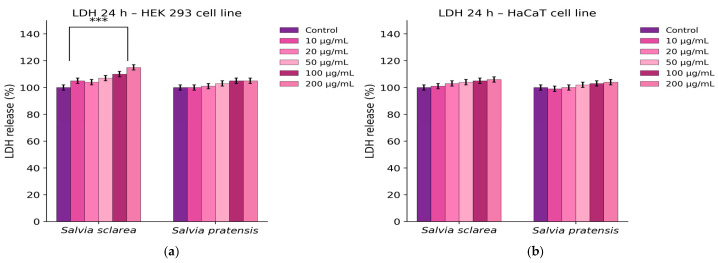
Lactate dehydrogenase (LDH) release in cells after 24 h treatment with *Salvia* extracts (10–200 µg/mL): (**a**) HEK 293 cells; (**b**) HaCaT cells. LDH levels are expressed as % of control (mean ± SD). Statistical analysis was performed using two-way ANOVA followed by Dunnett’s test (*p* < 0.05 vs. control) *** *p* < 0.001.

After treatment of HEK 293 and HaCaT cells with increasing concentrations of *S. sclarea* and *S. pratensis* extracts (10–200 µg/mL), no significant increase in lactate dehydrogenase (LDH) release was observed compared to the control, indicating that the extracts did not induce detectable cytotoxicity or membrane damage in the tested cell lines after 24 h, as indicates Figure 7.

**Figure 8:**
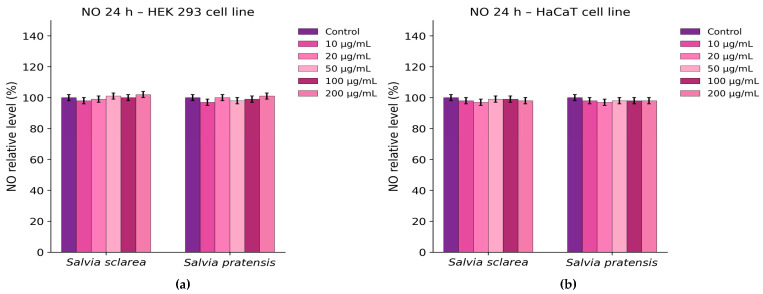
Nitric oxide (NO) production in cells after 24 h treatment with *Salvia* extracts (10–200 µg/mL): (**a**) HEK 293 cells; (**b**) HaCaT cells. NO levels are expressed as % of control (mean ± SD). Statistical analysis was performed using two-way ANOVA followed by Dunnett’s test (*p* < 0.05 vs. control).

Similarly, analysis of nitric oxide (NO) production showed no significant changes relative to untreated cells (Figure 8), suggesting that the extracts did not provoke oxidative or inflammatory responses under these conditions.

## 3. Discussion

The total phenolic content (TPC) determined for *S. sclarea* in the present study is consistent with previously reported values for this species, confirming hydroethanolic extracts as relevant sources of phenolic compounds [32]. Higher TPC values and stronger DPPH radical-scavenging activities have also been reported in the literature, with considerable variation depending on cultivar, geographical origin, and extraction conditions [1,33]. This variability suggests that antioxidant capacity is influenced not only by total phenolic content, but also by the qualitative phenolic profile and extract normalization. In this respect, the HPLC–PDA data obtained herein, showing the predominance of rosmarinic and protocatechuic acids in *S. sclarea*, support the observed antioxidant potential.

Although the phytochemistry of *S. sclarea* has been extensively investigated, *S. pratensis* remains comparatively underexplored despite its documented richness in phenolic compounds and biological activity. In the present study, *S. pratensis* hydroethanolic extracts exhibited pronounced antioxidant and antimicrobial effects, despite the species being characterized by a very low essential oil yield. HPLC–PDA analysis revealed a phenolic acid profile enriched in hydroxycinnamic acids, including caffeic, *p*-coumaric, and ferulic acids, which are known contributors to antioxidant and antimicrobial activity. In agreement with previous reports, the biological effects of *S. pratensis* are unlikely to depend exclusively on total phenolic content, but rather on the contribution of multiple classes of secondary metabolites and their possible synergistic interactions [1,24,27,34]. Accordingly, the favourable antioxidant performance and biocompatibility profile observed in this study most likely reflect the complexity of the phytochemical composition rather than the action of a single dominant constituent [27,35].

The GC–MS analysis of *S. sclarea* essential oil showed a predominance of oxygenated monoterpenes, in agreement with previous studies describing linalyl acetate and linalool as major constituents [13,36]. Although the overall qualitative profile was consistent with literature data, quantitative differences in the relative abundance of individual components were evident. Such variability has frequently been attributed to geographical origin, environmental conditions, plant organ, and extraction parameters [22,37]. In particular, diterpene compounds such as sclareol and its oxidation derivatives were not detected in the present sample, further underlining the influence of geographical origin and plant material on essential oil composition.

The antioxidant assays consistently showed stronger activity for *S. pratensis* than for *S. sclarea*. In the DPPH assay, the hydroethanolic extracts displayed pronounced radical-scavenging activity, with IC_50_ values of 3.16 µg/mL for *S. pratensis* and 6.67 µg/mL for *S. sclarea*. These results are in line with the phenolic acid profiles revealed by HPLC, particularly the presence of rosmarinic and caffeic acids, which are well known for their hydrogen-donating and electron-transfer properties. Compared with literature data, the IC_50_ values obtained in the present study fall within, or even below, the range commonly reported for *Salvia* species extracted with alcoholic or hydroethanolic solvents [1,38,39,40]. Nevertheless, direct comparisons should be interpreted cautiously, as DPPH activity is reported using different calculation approaches and assay conditions, including differences in radical concentration, solvent system, incubation time, and data processing [27]. As expected, vitamin C exhibited markedly stronger radical-scavenging activity than both plant extracts.

The FRAP assay supported the DPPH results, showing a markedly higher reducing capacity for *S. pratensis* than for *S. sclarea*. This trend is consistent with the higher phenolic richness of *S. pratensis*, especially in hydroxycinnamic acid derivatives, and agrees with previous reports describing positive associations between FRAP values and phenolic composition in *Salvia* species [41,42]. The ABTS assay confirmed the same ranking, with *S. pratensis* again showing stronger activity than *S. sclarea*. The higher IC_50_ values obtained in the ABTS assay relative to DPPH are most likely related to differences in radical type, reaction mechanism, and solvent compatibility, indicating that the antioxidant constituents of the extracts may react differently depending on the test system [43]. Taken together, the DPPH, FRAP, and ABTS results consistently indicate a superior antioxidant potential for *S. pratensis*, most likely associated with its phenolic composition.

The antimicrobial activity observed for both *Salvia* extracts is consistent with the well-documented bioactivity of the genus, which has been linked to phenolic diterpenes, triterpenic acids, and phenolic acids [44,45]. The MIC values obtained in the present study (312.5–2500 µg/mL) are higher than those reported for isolated compounds or essential oils, but fall within the range generally described for crude solvent extracts. This supports the view that antimicrobial activity in complex plant matrices results from additive or synergistic interactions among multiple phytochemicals. Notably, *S. pratensis* showed stronger antimicrobial activity than *S. sclarea* against most tested microorganisms, in agreement with recent reports highlighting the antimicrobial potential of phenolic-rich extracts from this species [24]. By contrast, although *S. sclarea* essential oil is widely recognized for its antimicrobial properties, non-volatile extracts often display only moderate activity, a pattern also observed here [13]. The lack of inhibition in the solvent control confirmed that the antimicrobial effects were attributable to intrinsic phytochemical constituents rather than solvent interference.

The observed biological activities are plausibly related to the structural features of phenolic acids such as rosmarinic and caffeic acids, whose hydroxyl groups favour radical scavenging, while their conjugated aromatic systems stabilize the resulting radicals [46]. In addition, as weak organic acids, such compounds may contribute to antimicrobial effects through membrane permeation and intracellular acidification. These mechanisms likely operate alongside the contribution of other constituents present in the extracts [47].

Chemical safety assessment demonstrated minimal contamination in the analysed samples. Pyrethroid residues were detected only in *S. sclarea*, most likely reflecting differences in cultivation practices or environmental exposure rather than intrinsic interspecific differences, and all concentrations remained below the relevant maximum residue limits [48]. Likewise, Cd and Pb were detected at low levels in both species. These findings indicate low contamination of the investigated plant material and support its suitability for further pharmacological consideration. Given that heavy metal accumulation in medicinal plants may vary considerably depending on environmental conditions, continued monitoring remains advisable [49,50,51].

The use of human cell lines represents an important step in assessing the safety profile of plant-derived extracts. In the present study, HEK293 and HaCaT cells were selected as complementary in vitro models commonly employed in biocompatibility and phytochemical research. HEK293 cells are widely used in toxicological and pharmacological studies because of their stable growth characteristics and sensitivity to bioactive compounds, making them suitable for evaluating the general cytotoxicity of natural products. Several studies on phytochemicals and herbal extracts have used HEK293 cells to assess cell viability, oxidative stress responses, and the potential toxicological effects of plant-derived compounds [52,53]. HaCaT cells, originally described by Boukamp et al., are a well-established immortalized human keratinocyte model that retains key features of normal epidermal differentiation and is widely used to study the effects of natural compounds on skin cells [54]. They are particularly relevant for evaluating the cytotoxic, antioxidant, and protective effects of plant-derived molecules in dermatological or topical research. In this context, the use of HaCaT cells is also supported by studies showing that *Salvia*-derived compounds, including salvianolic acid B, can protect keratinocytes against oxidative or UV-induced damage [55,56].

Therefore, the combined use of HEK293 and HaCaT cells provided complementary information on the biocompatibility of the tested extracts, allowing the assessment of both general cellular toxicity and skin-related biological relevance.

Thus, the in vitro safety evaluation further supported the favourable profile of the *Salvia* extracts. In HEK 293 cells, *S. sclarea* caused only a modest concentration-dependent decrease in viability at the highest tested concentrations, whereas *S. pratensis* showed minimal effects. In HaCaT keratinocytes, neither extract produced significant changes in viability. The absence of increased LDH release and nitric oxide production further indicated that the extracts did not induce detectable membrane damage or pro-inflammatory responses under the tested conditions. These findings are in agreement with previous reports describing low cytotoxicity of *Salvia* extracts toward non-transformed human cell lines [57,58]. One limitation of the present study is the absence of a dedicated inflammatory cell model, such as RAW 264.7 macrophages stimulated with lipopolysaccharide (LPS), which are commonly used for evaluating nitric oxide production. In the current work, the Griess assay was applied only as a preliminary indicator of nitrite levels in epithelial-derived cell lines (HaCaT and HEK293). Future studies will include appropriate inflammatory models and positive controls to further validate the biological activity of the *Salvia* extracts investigated.

Several other limitations of the present study should nevertheless be acknowledged, including the focus on selected classes of phytoconstituents, the potential influence of seasonal and geographical factors on composition, the use of a single extraction system, and the absence of in vivo safety validation. Future research should therefore aim at a more comprehensive phytochemical characterization, as well as the identification of the compounds primarily responsible for the observed biological activities. In this context, bioassay-guided fractionation and isolation approaches would be particularly valuable to correlate specific constituents with their biological effects. From an applied perspective, these species may also be considered for incorporation into advanced delivery systems designed for dermal or urinary tract applications.

Overall, the combined assessment of phytochemical composition, biological activity, chemical contamination, and in vitro biocompatibility provides a robust basis for evaluating the therapeutic potential of the investigated *Salvia* species. The results indicate that the antioxidant and antimicrobial properties of *S. sclarea* and especially *S. pratensis* are primarily associated with their intrinsic phytochemical composition, while their low levels of contaminants and limited cytotoxicity support their potential as promising candidates for further pharmaceutical development.

## 4. Materials and Methods

### 4.1. Plant Material and Preparation of the Extracts

The aerial parts of the plants were collected from Mihai Viteazu village, Călărași County, Romania (44°21′3″ N, 27°4′9″ E), during May–July 2025. Voucher specimens were deposited at the “Dimitrie Brândză” Botanical Garden, Bucharest, Romania, under accession numbers 409834 for *Salvia sclarea* L. and 409835 for *Salvia pratensis* L.

The aerial parts of *Salvia sclarea* and *Salvia pratensis* were extracted using a hydroethanolic solvent system (1 g of the dried material and 100 mL of ethanol/water, 50:50, *v*/*v*), by refluxing at 100 °C for 30 min on an electric water bath (Witeg Labortechnik, Wertheim, Germany). After extraction, the solvent was removed under reduced pressure using a Büchi Vacuum Pump V-700 rotary evaporator (Büchi, Uster, Switzerland). The remaining aqueous residue was subsequently frozen at −80 °C and then lyophilized to ensure the complete removal of water yielding the final dried hydroethanolic extracts.

The fine, powdered, dry extracts were kept in a glass vacuum desiccator. Prior to biological assays, the dried extracts were reconstituted in appropriate solvents according to the requirements of each experimental method.

### 4.2. Chemicals

All chemicals used in the experiments were of analytical grade. Folin–Ciocalteu reagent, gallic acid standard, vitamin C, ferrous sulfate, acetate buffer, 2,4,6-tripyridyl-s-triazine (TPTZ), 2,2′-azinobis(3-ethylbenzothiazoline-6-sulfonic acid) (ABTS), and potassium persulfate were purchased from Sigma-Aldrich (St. Louis, MO, USA). Ethanol, sodium carbonate, DPPH, hydrochloric acid, and ferric chloride hexahydrate were obtained from Merck (Darmstadt, Germany). Ultrapure water obtained from a Milli-Q purification system (Millipore, Bedford, MA, USA) was used throughout the experiments.

### 4.3. HPLC–PDA Identification and Quantification of Phenolic Acids

For sample preparation, plant material was extracted using an ultrasound-assisted extraction (UAE) method with 70% ethanol as the solvent. A precisely weighed amount of 0.1 g of finely powdered plant material was mixed with 10 mL of ethanol solution in a suitable container and subjected to ultrasonic treatment using a Bandelin Sonorex Digiplus DL 102 H ultrasound bath (Bandelin electronic GmbH & Co. KG, Berlin, Germany; power 100 W; frequency 35 kHz) for 20 min at 50 °C. The ultrasonic treatment facilitated the disruption of plant cell walls and enhanced the release of bioactive compounds into the solvent. After extraction, the solution was filtered through a 0.22 μm syringe filter with a WWPTFE membrane (Acrodisc, Pall Corporation, Port Washington, NY, USA) to remove solid residues prior to analysis [59,60].

Eight phenolic acids were used as reference standards: rosmarinic acid, caffeic acid, chlorogenic acid, p-coumaric acid, ferulic acid, protocatechuic acid, syringic acid, and vanillic acid. Stock solutions (1 mg/mL) were prepared in methanol and further diluted to obtain calibration standards in the range of 0.1–50 μg/mL. A volume of 10 μL was injected for both standards and samples.

HPLC analysis was performed using a Waters Acquity Arc system equipped with a photodiode array (PDA) detector and a QDa mass detector. Chromatographic separation was achieved on a CORTECS C18 column (4.6 × 50 mm, 2.7 μm particle size) maintained at 30 °C. The mobile phases consisted of water containing 0.01% formic acid (A) and acetonitrile containing 0.01% formic acid (B), delivered at a flow rate of 0.8 mL/min.

The gradient program started at 99% A and was held for 1 min, followed by a linear decrease to 70% A over 12 min. Subsequently, the composition was reduced to 20% A for column washing and maintained until 17.6 min. The system was then returned to the initial conditions (99% A) at 18.1 min and allowed to re-equilibrate until 21.1 min. Between injections, the column was equilibrated for 10 min to ensure reproducibility. Samples were kept at 8 °C throughout the analysis.

Quantification was performed at 265 nm for protocatechuic, vanillic, and syringic acids, and at 325 nm for chlorogenic, caffeic, p-coumaric, ferulic, and rosmarinic acids.

The HPLC–PDA method used in the present study was previously validated in accordance with ICH Q2(R2) guidelines, including evaluation of linearity, limits of detection (LOD), limits of quantification (LOQ), and precision. Detailed validation parameters are reported in our previous study [61].

### 4.4. Spectrophotometric Determination of Total Polyphenols

The total polyphenolic content (TPC) of the dried hydroethanolic extracts of S. sclarea and S. pratensis was determined using the Folin–Ciocalteu spectrophotometric method, with gallic acid as the reference standard [62,63]. Briefly, an aliquot of each extract solution was mixed with diluted Folin–Ciocalteu reagent, followed by the addition of sodium carbonate solution to obtain alkaline conditions. The reaction mixture was incubated at room temperature for 60 min, and the absorbance was measured at 765 nm using a UV–Vis spectrophotometer (UV-6300 PC, VWR International, Vienna, Austria).

Total polyphenolic content was calculated from a gallic acid calibration curve (Conc = 77.5789 × Abs, R^2^ = 0.9997; concentration range: 10–80 µg/mL), using a reagent blank consisting of all reagents except the sample, and expressed as milligrams of gallic acid equivalents per gram of dry extract (mg GAE/g dry extract). All measurements were performed in triplicate, and results are expressed as mean ± standard deviation (SD).

### 4.5. Hydrodistillation and GC-MS Analysis of the Essential Oil

Freshly ground aerial parts of *S. sclarea* and *S. pratensis* (150 g each) were subjected to hydrodistillation for 3 h using a glass Clevenger-type apparatus in a closed-loop system. The extraction was performed with 600 mL of distilled water according to the volumetric assay method described in the 10th edition of the European Pharmacopoeia. Each species was processed in triplicate to ensure reproducibility [64,65]. The essential oil yield was calculated as a percentage (% *v*/*w*), based on the volume of oil obtained relative to the mass of plant material, using the formula:

(1) $$yield\;\left(content\;of\;essential\;oil\right)=\frac{volume\;of\;Salvia\;essential\;oil\;\left(mL\right)}{mass\;of\;Salvia\;aerial\;parts\;\left(g\right)}\times 100$$

For further analysis, the essential oils were stored in amber vials in the fridge, at 4 °C, in the dark.

The chemical composition of the essential oils was analysed by gas chromatography–mass spectrometry (GC–MS). Analyses were carried out using a Thermo Electron Corporation Focus gas chromatograph coupled to a Thermo DSQII mass spectrometer (Thermo Scientific, Waltham, MA, USA) equipped with a split injector. Separation was achieved on a 30 m × 0.25 mm capillary column coated with Macrogol 20,000 (film thickness 0.25 µm; Ohio Valley, OH, USA). Helium was used as the carrier gas at a flow rate of 1.5 mL/min, and 1.0 µL of each sample was injected. The oven temperature was programmed from 65 °C to 200 °C over a 60 min period. The mass spectrometer was operated in electron ionization (EI) mode at 70 eV, with a mass scan range of *m*/*z* 40–400. Component quantification was performed by peak area normalisation. Compound identification was achieved by comparing the obtained mass spectra with reference spectra from the Wiley 8 and NIST 07 libraries, together with comparison of retention times and retention indices reported in the literature [61].

### 4.6. Chemical Safety Assessment

#### 4.6.1. Pesticide Residue Analysis

Pesticide residue analysis was performed as a complementary approach to support the chemical safety evaluation of *S. sclarea* and *S. pratensis*. All solvents and reagents were of analytical grade. Certified pesticide reference standards (purity ≥ 98%) for insecticides, fungicides, and herbicides commonly included in multiresidue analysis were used for calibration. Triphenyl phosphate (TPP; Sigma-Aldrich, Steinheim, Germany) was employed as an internal standard.

Sample preparation was carried out using a modified QuEChERS procedure in accordance with SR EN ISO 15662:2018 and the SANTE guidance document [66]. Briefly, homogenised plant samples were hydrated, spiked with the internal standard, and extracted with acetonitrile in the presence of a salt mixture (MgSO_4_, NaCl, sodium citrate dihydrate, and sodium citrate sesquihydrate). After centrifugation, clean-up was performed by dispersive solid-phase extraction (d-SPE) using PSA and MgSO_4_ sorbents.

Pesticide residues were analysed by liquid chromatography and gas chromatography coupled with tandem mass spectrometry (LC–MS/MS and GC–MS/MS) using triple-quadrupole instruments (Thermo Fisher Scientific, Waltham, MA, USA). Quantification was achieved using external calibration with matrix-matched standards. Calibration curves showed good linearity over the tested concentration ranges, with coefficients of determination (R^2^ ≥ 0.98). Quality control and method performance were evaluated in accordance with SANTE guidelines for pesticide residue analysis in food and feed [67].

#### 4.6.2. Determination of Heavy Metals

Cadmium (Cd) and lead (Pb) were determined from the same digest by graphite furnace atomic absorption spectrometry (GF-AAS) using a PerkinElmer AAnalyst 800 spectrometer (PerkinElmer, Waltham, MA, USA), equipped with an AS-800 autosampler (PerkinElmer, Waltham, MA, USA). Plant samples (*S. sclarea* and *S. pratensis*) were dried, homogenised, and subjected to dry ashing followed by acid dissolution, as previously described for plant-derived matrices [68,69]. Approximately 10 g of each sample was gradually heated, dried at 100 °C, and then calcined at 450 °C until complete mineralisation was achieved. After cooling, the ash was treated with hydrogen peroxide and reheated when necessary. The resulting residue was dissolved in hydrochloric acid, evaporated to dryness, and finally reconstituted in 0.1 mol/L nitric acid. Reagent blanks were prepared in parallel and processed using the same procedure.

Measurements of the absorbances were performed at analytical wavelengths of 228.8 nm for Cd and 283.3 nm for Pb.

Quantification was carried out by external calibration using aqueous standard solutions prepared from certified stock solutions. Calibration curves were established at five concentration levels within the ranges of 1–5 µg/L for Cd and 10–50 µg/L for Pb, obtained by automatic dilution of the corresponding working standard solutions. To minimise matrix interferences, ammonium dihydrogen phosphate and magnesium nitrate were used as matrix modifiers.

The analytical performance of the method was evaluated in terms of linearity, limits of detection (LOD), and limits of quantification (LOQ). All calibration curves showed good linearity, with correlation coefficients (R^2^ ≥ 0.995). The corresponding analytical performance parameters are summarised in Table 7. The obtained LOD and LOQ values confirmed the suitability of the method for the determination of trace levels of Cd and Pb in *Salvia* samples.

### 4.7. Biological Activity Determinations

#### 4.7.1. Antioxidant Activity

- DPPH Radical Scavenging Assay

The antioxidant activity of the dried hydroethanolic extracts of *S. sclarea* and *S. pratensis* was evaluated using the 2,2-diphenyl-1-picrylhydrazyl (DPPH) radical scavenging assay [33,62]. Stock solutions of the dried extracts were prepared in 50% ethanol and subsequently diluted to obtain final concentrations in the reaction mixture ranging from 5 to 50 µg/mL. An aliquot of 2 mL of each extract solution was mixed with 2 mL of DPPH solution (0.1 mM in 50% ethanol), incubated in the dark at room temperature for 30 min, and the absorbance was measured at 517 nm using a UV–Vis spectrophotometer (UV-6300 PC, VWR International, Vienna, Austria). The percentage of radical scavenging activity was calculated relative to the control, and IC_50_ values were determined by linear interpolation. A freshly prepared vitamin C solution (1 mg/mL in 50% ethanol) was used as a positive control under identical experimental conditions.

The percentage of DPPH radical scavenging activity was calculated using the equation:

(2) $$\%inhibition=\frac{A\text{\_}control-A\text{\_}sample}{A\text{\_}control}\times 100$$

where A_control represents the absorbance of the DPPH solution and A_sample corresponds to the absorbance of the extract–DPPH reaction mixture, both measured at 517 nm using ethanol as the blank. All experiments were performed in triplicate.

- Ferric Reducing Antioxidant Power (FRAP) Assay

The ferric reducing antioxidant power (FRAP) of the dried hydroethanolic extracts of *S. sclarea* and *S. pratensis* was determined according to a modified Benzie and Strain method [70]. The FRAP reagent was freshly prepared by mixing acetate buffer (300 mM, pH 3.6), 10 mM 2,4,6-tripyridyl-s-triazine (TPTZ) solution in 40 mM HCl, and 20 mM FeCl_3_·6H_2_O solution in a ratio of 10:1:1 (*v*/*v*/*v*).

An aliquot of extract solution was mixed with the FRAP reagent and incubated at 37 °C for 10 min in the dark. Absorbance was measured at 593 nm using a VWR UV-6300 PC spectrophotometer (UV-6300 PC, VWR International, Vienna, Austria). A calibration curve was constructed using ferrous sulfate (FeSO_4_·7H_2_O) as the standard, and results were expressed as µmol Fe^2+^ equivalents per gram of dry extract (µmol Fe^2+^/g d.e.). All determinations were performed in triplicate, and results are reported as mean ± SD.

- ABTS Radical Scavenging Assay

The antioxidant activity was evaluated using the ABTS radical cation decolorization assay according to the method of Re et al. [71]. The ABTS•^+^ radical was generated by reacting ABTS with potassium persulfate and incubating the mixture in the dark for 12–16 h at room temperature. Prior to analysis, the solution was diluted with ethanol to an absorbance of 0.70 ± 0.02 at 734 nm. For the assay, 0.05 mL of extract or standard solution was mixed with 4.95 mL of ABTS•^+^ solution, and the decrease in absorbance was measured at 734 nm after 6 min. Radical scavenging activity was expressed as IC_50_ values (final concentrations in the reaction mixture), and vitamin C was used as a reference antioxidant.

#### 4.7.2. Antimicrobial Activity

The antimicrobial activity of the *Salvia* extracts was evaluated against reference strains, including *Staphylococcus aureus* ATCC 25923, *Escherichia coli* ATCC 35218, *Pseudomonas aeruginosa* ATCC 27853, and *Candida albicans* ATCC 10231, obtained from the Deutsche Sammlung von Mikroorganismen und Zellkulturen (Braunschweig, Germany). The microbial strains were cultured on Plate Count Agar (PCA) at 37 ± 0.5 °C for 22 ± 2 h. Fresh microbial suspensions were prepared in sterile physiological saline from 24 h cultures and adjusted to 0.5 McFarland standard [37,68].

The minimum inhibitory concentration (MIC) was determined by the broth microdilution method using 96-well microplates. Serial twofold dilutions of the extracts were prepared in Nutrient Broth. Briefly, 100 µL of liquid medium was added to each well. In the first well, 100 µL of extract solution (10,000 µg/mL in DMSO) was added, followed by serial twofold dilutions by transferring 100 µL from one well to the next. After dilution, the final volume in each well was 100 µL. Subsequently, 10 µL of microbial suspension adjusted to 0.5 McFarland (1.5 × 10^8^ CFU/mL) was added to each well, resulting in a final inoculum of approximately 1.4 × 10^7^ CFU/mL per well.

Growth control wells (medium inoculated with microbial suspension) and sterility control wells (uninoculated medium) were included in each experiment. The plates were incubated at 37 °C for 24 h, and MIC values were determined by visual inspection as the lowest extract concentration at which no visible microbial growth was observed. To confirm microbial viability, 10 µL aliquots from each well were subcultured in triplicate onto PCA plates and incubated at 37 °C for an additional 24 h. The antimicrobial activity of the solvent (DMSO) was evaluated under the same experimental conditions using identical serial dilutions. Norfloxacin and nystatin were used as reference antimicrobial agents and tested under the same serial dilution conditions as the extracts.

### 4.8. In Vitro Biocompatibility and Cytotoxicity Assessment

The human keratinocyte cell line (HaCaT) and human embryonic kidney cells (HEK 293) were cultured in complete Dulbecco’s Modified Eagle Medium (DMEM; Gibco/Invitrogen, Carlsbad, CA, USA) supplemented with 10% fetal bovine serum (FBS; Gibco/Invitrogen, Carlsbad, CA, USA) and 1% antibiotic–antimycotic solution (Sigma-Aldrich, USA). Cells were maintained at 37 °C in a humidified atmosphere containing 5% CO_2_. Upon reaching appropriate confluence, cells were harvested and seeded into 96-well plates at a density of 3 × 10^4^ cells/mL. The cultures were subsequently treated with various concentrations of *Salvia* extracts (10–200 µg/mL) and incubated for 24 h. Untreated cells served as the negative control [72,73].

- Cell viability of HaCaT and HEK 293 cells was evaluated using the MTT assay [65]. Following treatment with various concentrations of *Salvia* extracts, the culture medium was removed, and the cells were incubated with MTT solution (1 mg/mL) for 2 h at 37 °C in a 5% CO_2_ atmosphere. The resulting formazan crystals were dissolved in isopropanol, and absorbance was measured at 595 nm using a FlexStation 3 microplate reader (Molecular Devices, San Jose, CA, USA).
- Cytotoxicity was assessed by measuring lactate dehydrogenase (LDH) release into the culture medium using the Cytotoxicity Detection Kit (LDH) (Roche, Basel, Switzerland). After 24 h of exposure to *Salvia* extracts (10–200 µg/mL), equal volumes of culture medium (50 µL) and reaction mixture were incubated for 20–30 min at room temperature in the dark. Absorbance was recorded at 490 nm using a microplate reader (Molecular Devices, USA).
- Nitric oxide (NO) production was quantified using the Griess reaction to evaluate potential pro-inflammatory effects of *S. sclarea* and *S. pratensis* on HaCaT and HEK 293 cells. After 24 h of treatment (10–200 µg/mL), culture supernatants (80 µL) were mixed with an equal volume of Griess reagent, and absorbance was measured at 540 nm. Results were expressed as percentages relative to the control.

Statistical analysis was performed using GraphPad Prism v. 8.0 (GraphPad Software, La Jolla, CA, USA). Data were analysed using two-way ANOVA followed by Dunnett’s multiple comparisons test. All experiments were performed in triplicate.

## 5. Conclusions

This study provides a comprehensive comparative assessment of the phytochemical composition, biological activities, and safety profiles of *Salvia sclarea* and *Salvia pratensis*. The results revealed clear species-specific differences in phenolic acid patterns and essential oil composition, with *S. pratensis* exhibiting higher total phenolic content and bioactive potential comparable to the widely cultivated *S. sclarea*. Both extracts demonstrated strong antioxidant capacity and moderate broad-spectrum antimicrobial activity against representative bacterial and fungal strains.

Chemical safety evaluation confirmed minimal contamination, with pesticide residues detected only in *S. sclarea* at levels below regulatory limits and low concentrations of heavy metals in both species. In vitro biocompatibility assays further indicated low cytotoxicity in normal human cell models, supporting the favorable biological profile of the investigated extracts under the tested conditions.

Overall, the integrated phytochemical, biological, and safety findings highlight *S. pratensis* as a promising yet underexplored species with potential applications as a natural source of antioxidant and antimicrobial compounds. These results emphasize the importance of integrating phytochemical characterization with chemical and biological safety assessment in the quality evaluation and sustainable valorization of medicinal plant resources. Future studies should focus on the isolation and characterization of the individual bioactive compounds responsible for the observed activities, as well as on in vivo validation and potential applications in pharmaceutical, nutraceutical, or functional plant-based products.

## Figures and Tables

**Table 1:** Identification and quantification of phenolic acids in *S. sclarea* and *S. pratensis* extracts by HPLC–PDA.

No.	Rt (min)	UV λmax (nm)	Compound	Subclass	*S. sclarea* μg/g d.w.	*S. pratensis* μg/g d.w.
1	3.70	265	Protocatechuic acid	Hydroxybenzoic acid	44.17 ± 1.59	2.72 ± 0.01
2	5.83	325	Chlorogenic acid	Hydroxycinnamic acid	F *	7.20 ± 0.35
3	6.11	265	Vanillic acid	Hydroxybenzoic acid	2.70 ± 0.07	4.71 ± 0.12
4	6.36	325	Caffeic acid	Hydroxycinnamic acid	11.35 ± 0.229	31.54 ± 0.36
5	6.64	265	Syringic acid	Hydroxybenzoic acid	F	3.363 ± 0.108
6	7.80	325	p-Coumaric acid	Hydroxycinnamic acid	12.491 ± 0.398	16.322 ± 0.511
7	8.54	325	Ferulic acid	Hydroxycinnamic acid	0.905 ± 0.007	8.198 ± 0.017
8	10.38	325	Rosmarinic acid	Hydroxycinnamic acid	181.296 ± 5.256	92.024 ± 3.875

* F: detected but not quantified (below LOQ).

**Table 2:** Comparative chemical composition of the essential oils of *S. sclarea* L. and *S. pratensis* L. determined by GC–MS analysis.

No.	Compound	RT (min)	RI (lit)	Chemical Class	*S.sclarea* (%)	*S.pratensis* (%)
1	D-Limonene	12.592	1024	Monoterpene hydrocarbon	9.32	–
2	Eucalyptol	12.860	1031	Oxygenated monoterpene	0.03	2.27
3	β-Linalool	27.21	1095	Oxygenated monoterpene	26.03	80.52
4	Linalyl acetate	27.36	1256	Oxygenated monoterpene	58.39	–
5	Caryophyllene	28.00	1418	Sesquiterpene hydrocarbon	–	5.81
6	cis-β-Copaene	31.33	1435	Sesquiterpene hydrocarbon	–	7.30
7	α-Terpineol	31.39	1186	Oxygenated monoterpene	4.37	–
8	Nerol acetate	32.287	1365	Oxygenated monoterpene	0.68	–
9	β-Acorenol	32.03	1502	Oxygenated sesquiterpene	–	1.26
10	Sabinene	32.78	969	Monoterpene hydrocarbon	–	2.84
11	Geranyl acetate	33.134	1379	Oxygenated monoterpene	1.18	–
	Total identified (%)				100.00	100.00

“–” = compound not detected. Individual components are listed in order of increasing retention time (RT). Relative content (%) was calculated based on peak area normalisation. RT = retention time. RI (lit) = retention index reported in the literature [31].

**Table 3:** Distribution of volatile compounds by chemical classes in the essential oils of *S. sclarea* and *S. pratensis*.

Chemical Class	*S. sclarea* (%)	*S. pratensis* (%)
Monoterpene hydrocarbons	9.32	2.84
Oxygenated monoterpenes	90.68	82. 79
Sesquiterpene hydrocarbons	–	13.11
Oxygenated sesquiterpenes	–	1.26

“–” indicates that no compounds were detected. Values represent the sum of the relative percentages (%) of compounds within each chemical class.

**Table 4:** Pesticide residue occurrence in *Salvia* samples analysed by GC–MS/MS and LC–MS/MS.

Sample	Plant Species	GC–MS/MS Results	LC–MS/MS Results *	Residue Status
S1	*S. sclarea*	Permethrin, cypermethrin (>LOQ *)	<LOQ *	Pesticide residues detected
S2	*S. pratensis*	<LOQ *	<LOQ *	No pesticide residues detected

* LOQ: 0.01 mg/kg.

**Table 5:** Quantified pyrethroid residues in *S. sclarea* determined by GC–MS/MS.

Pesticide	Concentration (mg/kg)	LOQ (mg/kg)	EU MRL (mg/kg) *
Permethrin (sum of isomers)	0.039	0.01	0.05 *
Cypermethrin (sum of isomers)	0.041	0.01	2 *

* MRLs according to Regulation (EC) No. 396/2005.

**Table 6:** Cadmium and lead contents in *Salvia* samples expressed as mean ± standard deviation (SD) of three independent determinations (*n* = 3).

Sample	Cd [mg/kg]	Pb [mg/kg]
*S. sclarea*	0.166 ± 0.003	0.010 ± 0.001
*S. pratensis*	0.004 ± 0.001	0.065 ± 0.002
EU maximum level (mg/kg)	0.20	0.30

**Table 7:** Analytical performance parameters of the GF-AAS method.

Metal	Concentration Range (µg/L)	R^2^	LOD (µg/L)	LOQ (µg/L)
Cd	1–5	0.9994	0.30	1.00
Pb	10–50	0.9983	3.00	10.00

## Data Availability

The original contributions presented in this study are included in the article. Further inquiries can be directed to the corresponding authors.

## References

[1] Luca S.V., Skalicka-Woźniak K., Mihai C.-T., Gradinaru A.C., Mandici A., Ciocarlan N., Miron A., Aprotosoaie A.C. (2023). Chemical Profile and Bioactivity Evaluation of *Salvia* Species from Eastern Europe. Antioxidants.

[2] Pârvu C. (2006). Universul Plantelor.

[3] Kumar A., Thakur R., Gautam R.D., Chauhan R., Kumar D., Yadav A., Singh S., Singh S. (2024). Multi-Environment Evaluation of Clary Sage (*Salvia sclarea* L.) Selections for Yield and Essential Oil Traits under Western Himalayan Conditions. J. Appl. Res. Med. Aromat. Plants.

[4] Yuce E., Yildirim N., Yildirim N.C., Paksoy M.Y., Bagci E. (2014). Essential Oil Composition, Antioxidant and Antifungal Activities of *Salvia sclarea* L. from Munzur Valley in Tunceli, Turkey. Cell. Mol. Biol..

[5] Mervić M., Bival Štefan M., Kindl M., Blažeković B., Marijan M., Vladimir-Knežević S. (2022). Comparative Antioxidant, Anti-Acetylcholinesterase and Anti-α-Glucosidase Activities of Mediterranean *Salvia* Species. Plants.

[6] Kuźma Ł., Kalemba D., Różalski M., Różalska B., Więckowska-Szakiel M., Krajewska U., Wysokińska H. (2009). Chemical Composition and Biological Activities of Essential Oil from *Salvia sclarea* Plants Regenerated in vitro. Molecules.

[7] Raafat K., Habib J. (2018). Phytochemical Compositions and Antidiabetic Potentials of *Salvia sclarea* L. Essential Oils. J. Oleo Sci..

[8] Gross M., Nesher E., Tikhonov T., Raz O., Pinhasov A. (2013). Chronic Food Administration of *Salvia sclarea* Oil Reduces Animals’ Anxious and Dominant Behavior. J. Med. Food.

[9] Faridzadeh A., Salimi Y., Ghasemirad H., Kargar M., Rashtchian A., Mahmoudvand G., Karimi M.A., Zerangian N., Jahani N., Masoudi A. (2022). Neuroprotective Potential of Aromatic Herbs: Rosemary, Sage, and Lavender. Front. Neurosci..

[10] Lopresti A.L. (2017). *Salvia* (Sage): A Review of Its Potential Cognitive-Enhancing and Protective Effects. Drugs R D.

[11] Fiore G., Nencini C., Cavallo F., Capasso A., Bader A., Giorgi G., Micheli L. (2006). In vitro antiproliferative effect of six Salvia species on human tumor cell lines. Phytother Res..

[12] Shakeel-u-Rehman R., Rah B., Lone S.H., Rasool R.U., Farooq S., Nayak D., Chikan N.A., Chakraborty S., Behl A., Mondhe D.M. (2015). Design and Synthesis of Antitumor Heck-Coupled Sclareol Analogues: Modulation of BH3 Family Members by SS-12 in Autophagy and Apoptotic Cell Death. J. Med. Chem..

[13] Kačániová M., Vukovic N.L., Čmiková N., Galovičová L., Schwarzová M., Šimora V., Kowalczewski P.Ł., Kluz M.I., Puchalski C., Bakay L. (2023). *Salvia sclarea* Essential Oil Chemical Composition and Biological Activities. Int. J. Mol. Sci..

[14] Sharifi-Rad M., Ozcelik B., Altın G., Daşkaya-Dikmen C., Martorell M., Ramírez-Alarcón K., Alarcón-Zapata P., Morais-Braga M.F.B., Carneiro J.N.P., Alves Borges Leal A.L. (2018). *Salvia* spp. Plants—From Farm to Food Applications and Phytopharmacotherapy. Trends Food Sci. Technol..

[15] Ulubelen A., Topcu G., Eriş C., Sönmez U., Kartal M., Kurucu S., Bozok-Johansson C. (1994). Terpenoids from *Salvia sclarea*. Phytochemistry.

[16] Laville R., Castel C., Filippi J.J., Delbecque C., Audran A., Garry P.P., Legendre L., Fernandez X. (2012). Amphilectane Diterpenes from *Salvia sclarea*: Biosynthetic Considerations. J. Nat. Prod..

[17] Piller V., Piller F., Cartron J.P. (1986). Isolation and Characterization of an N-Acetylgalactosamine Specific Lectin from *Salvia sclarea* Seeds. J. Biol. Chem..

[18] Wu A.M. (2005). Lectinochemical Studies on the Glyco-Recognition Factors of a Tn (GalNAcα1→Ser/Thr) Specific Lectin Isolated from the Seeds of *Salvia sclarea*. J. Biomed. Sci..

[19] Yalcin H., Ozturk I., Tulukcu E., Sagdic O. (2011). Effect of γ-Irradiation on Bioactivity, Fatty Acid Compositions and Volatile Compounds of Clary Sage Seed (*Salvia sclarea* L.). J. Food Sci..

[20] Acimovic M., Kiprovski B., Rat M., Sikora V., Popovic V., Koren A., Brdar-Jokanovic M. (2018). *Salvia sclarea*: Chemical Composition and Biological Activity. J. Agron. Technol. Eng. Manag..

[21] El-Gohary A., Amer H., Salama A., Wahba H., Khalid K. (2020). Characterization of the Essential Oil Components of Adapted *Salvia sclarea* L. (Clary Sage) Plant under Egyptian Environmental Conditions. J. Essent. Oil Bear. Plants.

[22] Gad H.A., Mamadalieva R.Z., Khalil N., Zengin G., Najar B., Khojimatov O.K., Al Musayeib N.M., Ashour M.L., Mamadalieva N.Z. (2022). GC-MS Chemical Profiling, Biological Investigation of Three *Salvia* Species Growing in Uzbekistan. Molecules.

[23] Tomou E.-M., Fraskou P., Dimakopoulou K., Dariotis E., Krigas N., Skaltsa H. (2024). Chemometric Analysis Evidencing the Variability in the Composition of Essential Oils in 10 *Salvia* Species from Different Taxonomic Sections or Phylogenetic Clades. Molecules.

[24] Srećković N., Mišić D., Gašić U., Matić S.L., Katanić Stanković J.S., Mihailović N.R., Monti D.M., D’Elia L., Mihailović V. (2022). Meadow Sage (*Salvia pratensis* L.): A Neglected Sage Species with Valuable Phenolic Compounds and Biological Potential. Ind. Crops Prod..

[25] Šulniūtė V., Pukalskas A., Venskutonis P.R. (2017). Phytochemical Composition of Fractions Isolated from Ten *Salvia* Species by Supercritical Carbon Dioxide and Pressurized Liquid Extraction Methods. Food Chem..

[26] Nagy G., Dobos A., Günther G., Yang M.H., Blunden G., Crabb T.A., Máthé I. (1998). Abietane diterpenoids from the roots of *Salvia pratensis*. Planta Med..

[27] Gruľová D., Baranová B., Eliašová A., Brun C., De Martino L., Caputo L., Poračová J., Nastišin Ľ., Fejér J., Elshafie H.S. (2025). *Salvia pratensis* L. Extracts as Potential Eco-Friendly Herbicides for Sustainable Agricultural Applications. Sci. Rep..

[28] Janicsák G., Zupko I., Nikolova M., Forgo P., Vasas A., Máthé I., Blunden G., Hohmann J. (2011). Bioactivity-Guided Study of Antiproliferative Activities of *Salvia* Extracts. Nat. Prod. Commun..

[29] Anačkov G., Božin B., Zorić L., Vukov D., Mimica-Dukić N., Merkulov L., Igić R., Jovanović M., Boža P. (2009). Chemical Composition of Essential Oil and Leaf Anatomy of *Salvia bertolonii* Vis. and *Salvia pratensis* L. (Sect. *Plethiosphace*, Lamiaceae). Molecules.

[30] Rzepa J., Wojtal L., Staszek D., Grygierczyk G., Labe K., Hajnos M., Kowalska T., Waksmundzka-Hajnos M. (2009). Fingerprint of Selected *Salvia* Species by HS-GC-MS Analysis of Their Volatile Fraction. J. Chromatogr. Sci..

[31] Adams R.P. (2007). Identification of Essential Oil Components by Gas Chromatography/Mass Spectrometry.

[32] Hudz N., Yezerska O., Shanaida M., Horčinová Sedláčková V., Wieczorek P.P. (2019). Application of the Folin–Ciocalteu Method to the Evaluation of *Salvia sclarea* Extracts. Pharmacia.

[33] Jasicka-Misiak I., Poliwoda A., Petecka M., Buslovych O., Shlyapnikov V., Wieczorek P. (2018). Antioxidant Phenolic Compounds in *Salvia officinalis* L. and *Salvia sclarea* L.. Ecol. Chem. Eng. S.

[34] Levaya Y., Atazhanova G., Gabe V., Badekova K. (2025). A Review of Botany, Phytochemistry, and Biological Activities of Eight *Salvia* Species Widespread in Kazakhstan. Molecules.

[35] Ravlić M., Baličević R., Lisjak M., Vinković Ž., Ravlić J., Županić A., Svitlica B. (2025). Allelopathic Effect of *Salvia pratensis* L. on Germination and Growth of Crops. Crops.

[36] Ovidi E., Laghezza Masci V., Zambelli M., Tiezzi A., Vitalini S., Garzoli S. (2021). *Laurus nobilis*, *Salvia sclarea* and *Salvia officinalis* Essential Oils and Hydrolates: Evaluation of Liquid and Vapor Phase Chemical Composition and Biological Activities. Plants.

[37] Filipović S., Vasić T., Paдулoвич H., Stanojević I., Jevremović D. (2021). Detailed GC-MS Analysis of the *Salvia sclarea* L. Essential Oil and the First In Vitro Antifungal Activity Assessment against the Crop Pathogen *Colletotrichum acutatum* J.H. Simmonds. J. Essent. Oil Res..

[38] Durling N., Catchpole O., Grey J., Webby R., Mitchell K., Foo L., Perry N. (2007). Extraction of Phenolics and Essential Oil from Dried Sage (*Salvia officinalis*) Using Ethanol–Water Mixtures. Food Chem..

[39] Grzegorczyk-Karolak I., Kiss A.K. (2018). Determination of the Phenolic Profile and Antioxidant Properties of *Salvia viridis* L. Shoots: A Comparison of Aqueous and Hydroethanolic Extracts. Molecules.

[40] Quradha M.M., Duru M.E., Kucukaydin S., Tamfu A.N., Iqbal M., Bibi H., Khan R., Ceylan O. (2024). Comparative Assessment of Phenolic Composition Profile and Biological Activities of Green Extract and Conventional Extracts of *Salvia sclarea*. Sci. Rep..

[41] Hrebień-Filisińska A.M., Bartkowiak A. (2021). Antioxidative Effect of Sage (*Salvia officinalis* L.) Macerate as “Green Extract” in Inhibiting the Oxidation of Fish Oil. Antioxidants.

[42] Iacopetta D., Ceramella J., Scumaci D., Catalano A., Sinicropi M.S., Tundis R., Alcaro S., Borges F. (2023). An Update on Recent Studies Focusing on the Antioxidant Properties of *Salvia* Species. Antioxidants.

[43] Chaves N., Santiago A., Alías J.C. (2020). Quantification of the antioxidant activity of plant extracts: Analysis of sensitivity and hierarchization based on the method used. Antioxidants.

[44] Kamatou G.P.P., Makunga N.P., Ramogola W.P., Viljoen A.M. (2008). South African *Salvia* species: A review of biological activities and phytochemistry. J. Ethnopharmacol..

[45] Pavić V., Jakovljević M., Molnar M., Jokić S. (2019). Extraction of Carnosic Acid and Carnosol from Sage (*Salvia officinalis* L.) Leaves by Supercritical Fluid Extraction and Their Antioxidant and Antibacterial Activity. Plants.

[46] Heleno S.A., Martins A., Queiroz M.J.R.P., Ferreira I.C.F.R. (2015). Bioactivity of phenolic acids: Metabolites versus parent compounds: A review. Food Chem..

[47] Kumar N., Goel N. (2019). Phenolic acids: Natural versatile molecules with promising therapeutic applications. Biotechnol. Rep..

[48] Kowalska G. (2020). Pesticide Residues in Some Polish Herbs. Agriculture.

[49] Kiliçel F., Karapınar H., Uğuz A. (2017). Determination of Some Heavy Metal Concentrations of Sage Tea with FAAS. Int. J. Second. Metab..

[50] European Commission (2005). Regulation (EC) No 396/2005 of the European Parliament and of the Council of 23 February 2005 on maximum residue levels of pesticides in or on food and feed of plant and animal origin and amending Council Directive 91/414/EEC. Off. J. Eur. Union.

[51] Dobrikova A.G., Apostolova E.L., Hanć A., Yotsova E., Borisova P., Sperdouli I., Adamakis I.-D.S., Moustakas M. (2021). Cadmium Toxicity in *Salvia sclarea* L.: An Integrative Response of Element Uptake, Oxidative Stress Markers, Leaf Structure and Photosynthesis. Ecotoxicol. Environ. Saf..

[52] Atanasov A.G., Waltenberger B., Pferschy-Wenzig E.-M., Linder T., Wawrosch C., Uhrin P., Temml V., Wang L., Schwaiger S., Heiss E.H. (2015). Discovery and resupply of pharmacologically active plant-derived natural products: A review. Biotechnol. Adv..

[53] Atanasov A.G., Zotchev S.B., Dirsch V.M., Supuran C.T., International Natural Product Sciences Taskforce (2021). Natural products in drug discovery: Advances and opportunities. Nat. Rev. Drug Discov..

[54] Boukamp P., Petrussevska R.T., Breitkreutz D., Hornung J., Markham A., Fusenig N.E. (1988). Normal keratinization in a spontaneously immortalized aneuploid human keratinocyte cell line. J. Cell Biol..

[55] Hamidpour M., Hamidpour R., Hamidpour S., Shahlari M. (2014). Chemistry, pharmacology, and medicinal property of sage (*Salvia*) to prevent and cure illnesses such as obesity, diabetes, depression, dementia, lupus, autism, heart disease, and cancer. J. Tradit. Complement. Med..

[56] Zhang Q.J., Luo X., Zheng Y.W., Zheng J.Q., Wu X.Y., Wang S.M., Shi J. (2025). Salvianolic acid B exerts antiphotoaging effect on ultraviolet B-irradiated human keratinocytes by alleviating oxidative stress via SIRT1 protein. Chin. J. Integr. Med..

[57] Srećković N.Z., Nedić Z.P., Monti D.M., D’Elia L., Dimitrijević S.B., Mihailović N.R., Katanić Stanković J.S., Mihailović V.B. (2023). Biosynthesis of Silver Nanoparticles Using *Salvia pratensis* L. Aerial Part and Root Extracts: Bioactivity, Biocompatibility, and Catalytic Potential. Molecules.

[58] Michalak M., Zagórska-Dziok M., Żarnowiec P., Ostróżka-Cieślik A., Bocho-Janiszewska A., Stryjecka M., Dobros N., Kostrzewa D., Paradowska K. (2025). Evaluation of *Salvia yangii* Extract as a Promising Protective Raw Material Applied Topically to the Skin. Molecules.

[59] Bejenaru L.E., Biţă A., Mogoşanu G.D., Segneanu A.-E., Radu A., Ciocîlteu M.V., Bejenaru C. (2024). Polyphenols Investigation and Antioxidant and Anticholinesterase Activities of *Rosmarinus officinalis* L. Species from Southwest Romania Flora. Molecules.

[60] Gălăţanu M.L., Panţuroiu M., Ordeanu V., Neagu R., Gavriloaia R.M., Aurică S.N., Costache G.M. (2026). Revealing the Bioactive Potential of Romanian Wild Hop Cones: An Integrative Chemical, Antimicrobial, and Antibiofilm Activity and In Silico Docking Analysis. Molecules.

[61] Golu R.M., Bejenaru L.E., Biţă A., Bejenaru C., Segneanu A.-E., Ciocîlteu M.V., Blendea A., Neamţu J., Mogoşanu G.D. (2026). Standardization of Romanian *Galeopsis tetrahit* Leaf Extract in Verbascoside Using a Validated UHPLC–PDA Method. Plants.

[62] Panţuroiu M., Gălăţanu M.L., Manea C.E., Popescu M., Sandulovici R.C., Pănuş E. (2025). From Chemical Composition to Biological Activity: Phytochemical, Antioxidant, and Antimicrobial Comparison of *Matricaria chamomilla* and *Tripleurospermum inodorum*. Compounds.

[63] Gălăţanu M.L., Panţuroiu M., Cima L.M., Neculai A.M., Pănuş E., Bleotu C., Enescu C.M., Mircioiu I., Gavriloaia R.M., Aurică S.N. (2025). Polyphenolic Composition, Antioxidant Activity, and Cytotoxic Effect of Male Floral Buds from Three *Populus* Species Growing in the South of Romania. Molecules.

[64] Panţuroiu M., Gălăţanu M.L., Cristache R.E., Truţă E. (2025). Characterisation of nutraceutical fatty acids and amino acids in the buds of *Populus nigra*, *Populus alba*, and *Populus* × *euramericana* by gas chromatography. J. Sci. Arts.

[65] European Directorate for the Quality of Medicines HealthCare of the Council of Europe (2019). European Pharmacopoeia.

[66] (2018). Foods of Plant Origin—Multimethod for the Determination of Pesticide Residues Using GC- and LC-Based Analysis Following Acetonitrile Extraction/Partitioning and Clean-Up by Dispersive SPE-Modular QuEChERS-Method.

[67] European Commission (2021). Guidance Document on Analytical Quality Control and Method Validation Procedures for Pesticide Residues Analysis in Food and Feed (SANTE/11312/2021).

[68] Sandulovici R.C., Gălăţanu M.L., Cima L.M., Panus E., Truţă E., Mihăilescu C.M., Sârbu I., Cord D., Rîmbu M.C., Anghelache Ş.A. (2024). Phytochemical Characterization, Antioxidant, and Antimicrobial Activity of the Vegetative Buds from Romanian Spruce, *Picea abies* (L.) H. Karst. Molecules.

[69] Cima L., Sandulovici R., Stanciu G., Cristache R., Truță E. (2025). Chemical Profiling and Antioxidant Assessment of Two *Coffea arabica* Varieties. J. Sci. Arts.

[70] Benzie I.F.F., Strain J.J. (1996). The Ferric Reducing Ability of Plasma (FRAP) as a Measure of “Antioxidant Power”: The FRAP Assay. Anal. Biochem..

[71] Re R., Pellegrini N., Proteggente A., Pannala A., Yang M., Rice-Evans C. (1999). Antioxidant activity applying an improved ABTS radical cation decolorization assay. Free Radic. Biol. Med..

[72] Stancu A.I., Dițu L.M., Oprea E., Ficai A., Badea I.A., Buleandră M., Brîncoveanu O., Mirea A.G., Voicu S.N., Musuc A.M. (2025). New Antimicrobial Gels Based on Clove Essential Oil–Cyclodextrin Complex and Plant Extracts for Topical Use. Gels.

[73] Mosmann T. (1983). Rapid colorimetric assay for cellular growth and survival: Application to proliferation and cytotoxicity assays. J. Immunol. Methods.

